# Sec61β, a subunit of the Sec61 protein translocation channel at the Endoplasmic Reticulum, is involved in the transport of Gurken to the plasma membrane.

**DOI:** 10.1186/1471-2121-10-11

**Published:** 2009-02-18

**Authors:** Anshuman Kelkar, Bernhard Dobberstein

**Affiliations:** 1 Zentrum für Molekulare Biologie Universitat Heidelberg, Im Neuenheimer Feld 282, Heidelberg 69120, Germany; 2 The Rockefeller University and Howard Hughes Medical Institute, 1230 York Avenue, New York, NY 10065, USA

## Abstract

**Background:**

Protein translocation across the membrane of the Endoplasmic Reticulum (ER) is the first step in the biogenesis of secretory and membrane proteins. Proteins enter the ER by the Sec61 translocon, a proteinaceous channel composed of three subunits, α, β and γ. While it is known that Sec61α forms the actual channel, the function of the other two subunits remains to be characterized.

**Results:**

In the present study we have investigated the function of Sec61β in *Drosophila melanogaster*. We describe its role in the plasma membrane traffic of Gurken, the ligand for the Epidermal Growth Factor (EGF) receptor in the oocyte. Germline clones of the mutant allele of Sec61β show normal translocation of Gurken into the ER and transport to the Golgi complex, but further traffic to the plasma membrane is impeded. The defect in plasma membrane traffic due to absence of Sec61β is specific for Gurken and is not due to a general trafficking defect.

**Conclusion:**

Based on our study we conclude that Sec61β, which is part of the ER protein translocation channel affects a post-ER step during Gurken trafficking to the plasma membrane. We propose an additional role of Sec61β beyond protein translocation into the ER.

## Background

Translocation of proteins across the membrane of the Endoplasmic Reticulum (ER) is the first step in the biogenesis of secretory and membrane proteins. The nascent polypeptide chains of these proteins enter the ER via a proteinaceous channel called the Sec61p complex and gain access to the secretory pathway. If the mature protein does not possess specific signals for retention in the ER the protein exits the ER and is transported to the Golgi complex. The protein then moves along the Golgi complex till it reaches the trans-Golgi region. From the trans-Golgi region the protein is either transported to endocytic organelles or to the plasma membrane.

The Sec61 translocon is a heterotrimer constituted by α, β and γ subunits. According to the crystal structure of the SecY complex from *Methanococcus jannaschii*, which is presumed to represent the fundamental structure of the Sec61 protein translocation channel at the ER, the α subunit, forms the actual channel. The β and the γ subunits are associated with the channel on the periphery and are in contact with the lipid bilayer [1]. Data obtained from *in-vitro *experiments using liposomes reconstituted with individual components of the Sec61p channel indicate that Sec61α and γ are necessary for the protein translocation per-se [2]. Sec61β, on the other hand, is dispensable [2]. In accordance with the *in-vitro *data, in *S. cerevisiae *mutating α and γ subunits of Sec61p is lethal whereas mutating Sec61β results only in a mild temperature sensitive phenotype [3,4].

In contrast to the observations in *S. cerevisiae*, Sec61β is essential in *Drosophila melanogaster*; embryos homozygous for an allele with a P-element insertion before the transcription start site (*sec61β*^*P1*^) die at the end of embryogenesis, at stage 17 [5]. The *sec61β*^*P1 *^allele was used for generation of the maternal germline clones causing depletion of Sec61β protein from the ovaries. These flies produced embryos that died before stage 17 with the dorsal appendages completely or partially fused [5]. This dorsal appendage phenotype indicates perturbations in the dorsal-ventral axis with increased ventralization of the oocyte. Mutations that inhibit the EGF receptor signalling during oogenesis are known to cause this phenotype [6]. The only known EGF receptor ligand in the oocyte is Gurken. Gurken protein is synthesized in the oocyte but signals to the EGF receptor on the surrounding follicle cells [7]. Asymmetric signalling by Gurken to the follicle cells in the oocyte constitutes the first step towards formation of the dorsal-ventral axis in the embryo [7]. This asymmetric signalling is initiated when the Gurken mRNA is exclusively localized to the posterior end of the oocyte during early stages of oogenesis and to the anterior-dorsal end of the oocyte during the later stages [7]. Local translation of the mRNA results in synthesis of Gurken as a type I membrane protein in the ER. Like other members of the EGFR ligand family, transport of Gurken from the ER to the plasma membrane is highly regulated. An ER resident chaperone, Star, mediates the exit of Gurken from the ER [8]. During the transport through the secretory pathway to the plasma membrane Gurken protein is cleaved within the trans-membrane region by action of a member of the Rhomboid (Rho) family of intra-membrane proteases, called Brother of Rhomboid [8,9]. Cornichon ensures packaging of the Gurken protein in specialized COPII vesicles [10]. The soluble protein is secreted from the oocyte and interacts with the cognate receptor in the neighbouring follicle cells [8].

In this study, we investigated the possibility that the dorsal appendage fusion phenotype observed in the embryos from the *sec61β*^*P1 *^germline clones is due to changes in the biosynthesis of Gurken. We analysed the localization of Gurken mRNA and Gurken protein in wild type and sec61β mutant egg chambers. We found that during different stages of oogenesis depletion of Sec61β from the oocytes results in reduced levels of Gurken at the plasma membrane and in the surrounding follicle cells. We also observed that reduced amounts of Sec61β protein does not affect the ER translocation of Gurken, majority of the Gurken protein can enter the ER and is successfully transported till the Golgi complex. Transport of Gurken protein beyond the Golgi complex is affected by lack of Sec61β. This defect seems to be specific for Gurken since transport of a control protein is not affected. We propose that Sec61β plays an essential role during later stages of Gurken transport to the plasma membrane.

## Results

### Expression of Sec61β in wild type and *sec61β*^*P1 *^germline clones

**Figure 1:**
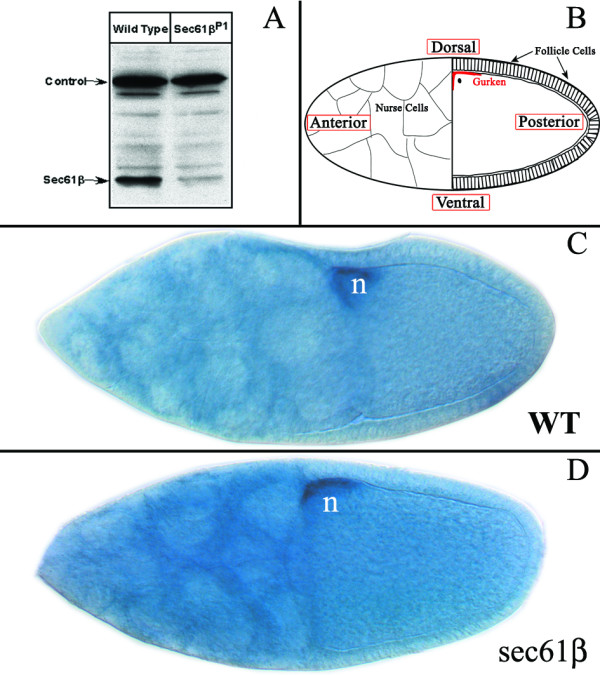
**Sec61β protein and Gurken mRNA in ovaries from wild type and *sec61β*^*P1 *^germline clones**. A) Ovaries were dissected from flies and lysed in buffer containing 2% SDS. Western blotting was performed using Sec61β antibody. A cross-reactive band serves as a loading control. B) Schematic representation of a stage 9–10 egg chamber with the oocyte abutting the 15 nurse cells and surrounded by a layer of somatic follicle cells. The dorsal/anterior corner is depicted in dark red. C-D) *Drosophila *egg chambers were processed for *gurken *RNA in-situ hybridization in wild type oocytes, C) and in oocytes mutant for Sec61β, D). n represents the oocyte nucleus.

The *sec61β*^*P1 *^allele has been reported to be a probable loss-of-function allele [5]. In order to characterise the allele in greater detail we generated an antibody against the N-terminus of Sec61β. This antibody was used for western blot analysis using protein extracts from wild type ovaries and ovaries derived from female germline homozygous for *sec61β*^* P1 *^(Fig. 1A). The amount of Sec61β protein is significantly reduced in ovaries derived from homozygous mutant clones as compared to the wild type ovaries. The small amount of Sec61β protein observed in *sec61β*^* P1 *^homozygous clones most likely represents Sec61β in follicle cells. Follicle cells do not originate from the germ cell lineage and should retain normal amounts of Sec61β [11].

### Localization of the Gurken mRNA is unaffected in the germline clones of Sec61β

The germline clones of the *sec61β*^*P1 *^allele generated embryos with fused dorsal appendages indicating perturbations in the dorsal-ventral axis [5]. The observed phenotype is strongly reminiscent of the dorsal-ventral axis defect found in the Gurken mutations. Asymmetric localization of the Gurken mRNA at the anterior-dorsal end of the ooycte is the first step towards establishment of the dorsal-ventral axis [12]. It has been proposed that the Gurken mRNA is closely associated with the ER and an ER protein could anchor the mRNA at the anterior-dorsal end of the oocyte [13].

We therefore examined the localization of Gurken mRNA in the stage 10 egg chambers by *in situ *hybridisation. In wild type oocytes Gurken mRNA localizes to the anterior-dorsal end of the oocyte in close proximity to the oocyte nucleus as indicated in the cartoon (Fig. 1B and 1C). In sec61β mutant oocytes Gurken mRNA similarly localizes to the anterior-dorsal end of the oocyte (Fig 1C and 1D). Thus, the *sec61β*^*P1*^germline clones show no apparent difference in the localization of Gurken mRNA in oocytes.

### Germline clones of *sec61β*^*P1 *^allele show reduced levels of Gurken protein at the plasma membrane of the oocyte

Since the mRNA localization of Gurken was not affected by lack of Sec61β, we investigated whether lack of Sec61β affects the translation of Gurken mRNA or events in the transport of Gurken protein to the plasma membrane.

**Figure 2:**
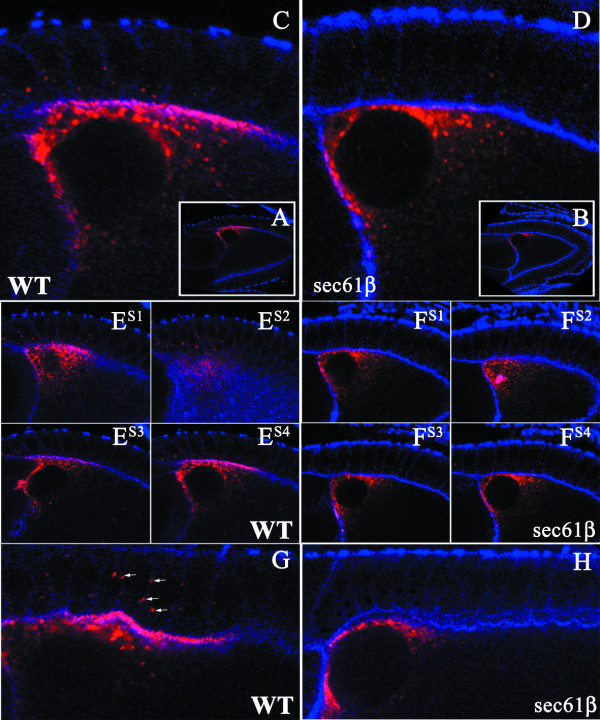
**Localization of Gurken Protein in Stage 10 Egg Chambers of Wild type and *sec61β*^*P1 *^Germline Clones**. *Drosophila *egg chambers of the indicated genotype, wild type, (WT) or egg chambers mutant for sec61β, (sec61β), stained for actin using labelled Phalloidin (blue) and Gurken (red). A-B) Low magnification images of wild type (A) and sec61β mutant egg chamber (B) at stage 10; Gurken appears at the anterior-dorsal position. C-D) Magnification of the anterior-dorsal region of wild type and sec61β mutant oocytes. Gurken co-localizes with actin in the wild type oocytes (C), where as the sec61β mutant oocytes do not show this co-localization (D). E^S1-S4^-F^S1-S4^) Optical sections of the egg chambers as shown in C and D, correspond to either wild type egg chambers (E^S1-S4^) or sec61β mutant egg chambers (F^S1-S4^). G-H) High magnification images of oocytes showing the region of the follicle cells from wild type (G) and sec61β mutant egg chambers (H). Gurken is seen in dot like structures within the follicle cells in the wild type egg chambers (indicated by arrows), these structures are absent in the mutant egg chambers.

We performed immuno-fluorescence analysis on wild type egg chambers or germline clones of *sec61β*^*P1 *^using a monoclonal antibody generated using the N-terminal extra-cellular domain of the Gurken protein (Developmental Studies Hybridoma Bank, [14]). The egg chambers were also stained with phalloidin, which binds actin localized below the plasma membrane and serves to delineate the plasma membrane. In stage 10 wild type oocytes Gurken protein localizes at the anterior-dorsal part of the oocyte in close proximity to the oocyte nucleus in precisely the same region as the mRNA (Fig. 2A). The mutant stage 10 oocytes also stains positive for Gurken, with the protein localized in the anterior-dorsal end of the oocyte (Fig. 2B). Upon analysis of the anterior-dorsal part of the oocytes at higher magnification we observed Gurken at two different locations in the oocyte, in punctuate structures in the cytoplasm between the plasma membrane and the nucleus and at the plasma membrane at the anterior-dorsal end of the oocyte, co-localizing with actin, directly opposite the follicle cells or the nurse cells (Fig. 2C). In the oocytes derived from the germline clones of *sec61β*^* P1 *^the cytoplasmic pool of Gurken protein was observed at the anterior-dorsal part of the oocyte in punctuate structures similar to the wild type oocytes but the Gurken protein amount at the plasma membrane was drastically reduced (Fig. 2D). Reduction in Gurken amount occurs in the part of the plasma membrane that is in direct apposition to the follicle cells and the nurse cells (Fig. 2D). To rule out the possibility that the observed changes in Gurken localization observed were due to differences in the focal plane in view, we analysed series of optical sections from different depths of the wild type oocytes (Fig. 2E^S1-S4^) and the mutant oocyte (Fig. 2F^S1-S4^). In all cases Gurken protein is excluded specifically from the plasma membrane.

We also observed staining for Gurken protein in distinct speckles inside the follicle cells at the anterior-dorsal end of the oocytes (Fig. 2G). These speckles most likely represent the protein that has been internalized by the follicle cells. In *sec61β*^*P1 *^germline clones on the other hand, Gurken staining in the follicle cells is rarely observed (Fig. 2H). Taken together, these results demonstrate that egg chambers from the *sec61β*^*P1 *^germline clones have reduced levels of Gurken protein at the plasma membrane of the oocyte and in the surrounding follicle cells.

### Gurken protein is also mis-localized during early oogenesis

**Figure 3:**
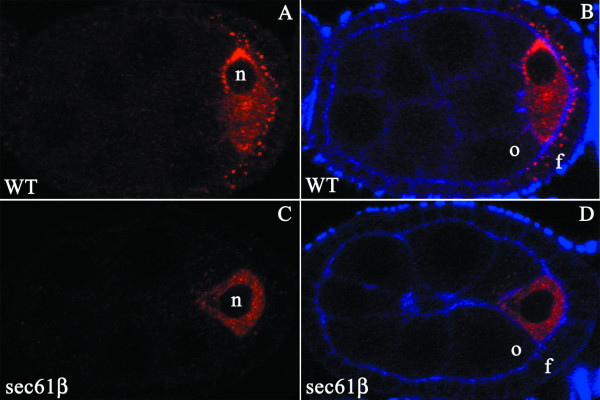
**Localization of Gurken Protein in Egg Chambers of Wild type and *sec61β*^*P1 *^Germline Clones during earlier stages of Oogenesis**. *Drosophila *egg chambers of the indicated genotype, wild type, (WT) or egg chambers mutant for sec61β, (sec61β), stained for actin using labelled Phalloidin (blue) and Gurken (red). A -B) Staining for Gurken protein (A) in the stage 6 oocyte from wild type, co-staining with actin (B) to delineate the follicle cells. C-D) Staining for Gurken protein (C) in sec61β mutant oocytes shows protein distributed all over the oocyte, co-staining with actin to delineate the follicle cells. f, indicates the follicle cells, o, the oocyte and n, the oocyte nucleus.

Gurken protein signals to the EGF receptor on the follicle cells at two different stages of oogenesis. During late oogenesis (stage 10–11) Gurken signals to follicle cells at the anterior-dorsal end of the oocyte. During earlier stages of oogenesis (stages 6–9) the oocyte nucleus and the Gurken mRNA are localized at the posterior part of the oocyte with Gurken protein signalling to the posterior follicle cells [12]. In order to investigate the localization of Gurken during early oogenesis we co-stained egg chambers during stage 6–9 of oogenesis with the anti-Gurken antibody and with phalloidin. The Gurken protein in the wild type egg chambers is localized in punctuate cytoplasmic structures towards the posterior part of the oocyte (Fig 3A and 3B). We also observe Gurken staining in the posterior follicle cells. In the oocytes derived from *sec61β*^*P1 *^germline clones we observe Gurken in the oocyte cytoplasm similar to wild type oocytes (Fig 3C and 3D) but not in the posterior follicle cells (Fig 3D). Thus, the Gurken trafficking defect is also observed during early stages of oogenesis.

### The general structure and function of ER remains unaffectedin *sec61β *mutant oocytes

**Figure 4:**
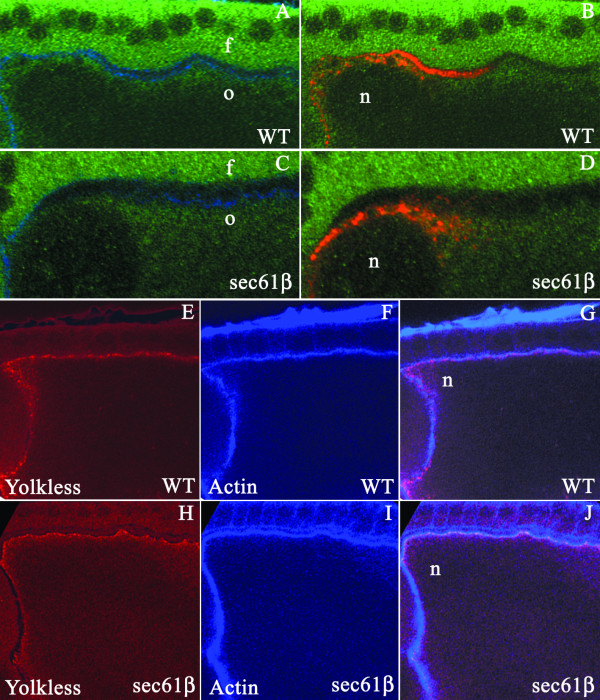
**Analysis of ER structure and function in sec61β mutant oocytes**. A-D, *Drosophila *egg chambers of the indicated genotype, wild type, (WT) or egg chambers mutant for sec61β, (sec61β), stained for actin using labelled Phalloidin (blue), Gurken (red) and Boca (green). A and C) In oocytes from stage 10 egg chambers Boca stains a diffuse region below the plasma membrane that marks the ER in both the wild type (A) and the sec61β^*P1*^germline clones (C). B and D) Gurken staining is observed either along the plasma membrane or in distinct punctate structures (B) or Gurken is excluded from the plasma membrane and is present exclusively in the cytoplam (D). f, indicates the follicle cells, o, the oocyte and n, the oocyte nucleus. E-J, *Drosophila *egg chambers of the indicated genotype, wild type, (WT) or egg chambers mutant for sec61β, (sec61β), stained for actin using labelled Phalloidin (blue) and Yolkless (red). In wild type egg chambers (E) or egg chambers mutant for sec61β (H) Yl is at a peripheral location in the oocyte. Co-staining with phalloidin (F and I) shows that both in wild type oocytes and oocytes from the germline clones Yl co-localizes at the plasma membrane (G and J). n indicates the position of the nucleus.

Gurken is a type I membrane protein and the presence of the signal sequence suggests that the protein is most likely co-translationally translocated into the ER and transported along the secretory pathway to reach the plasma membrane. To investigate if the mislocalization of Gurken was due to a general impairment in structure and function of ER, we co-stained wild type and sec61β mutant egg chambers with the Gurken antibody and an antibody raised against the Boca protein that has been previously characterised as an ER resident protein in oocytes [15]. In wild type egg chambers during stage 9–10 of oogenesis we observe a diffused staining for Boca in a region below the plasma membrane throughout the oocyte (Figure 4A). Oocytes derived from the *sec61β*^*P1 *^germline clones show a very similar staining, suggesting that the overall organization of the ER remains largely unaffected by lack of Sec61β(Fig. 4C). Localization of Gurken in the wild type and the sec61β mutant oocytes is as observed previously (Fig 4B,D, and Fig 2).

The Sec61 translocon translocates a variety of secretory and membrane proteins into the ER. The reduction in the Gurken amounts at the plasma membrane in the germline clones of *sec61β*^*P1 *^could be due to a general defect in the plasma membrane trafficking. In order to investigate this possibility we examined the localization of another plasma membrane protein of the oocyte, Yolkless (Yl). Yl is a type I membrane protein expressed by the oocytes for the uptake of vitellogenins and yolk proteins during oogenesis [16]. We co-stained wild type and mutant oocytes for Yolkless (Yl) and actin. Upon staining we observed that Yl is localized to the oocyte plasma membrane in both the wild type egg chambers (Fig.4E–G) and *sec61β*^*P1 *^homozygous clones (Fig.4H–J) during stage 9–10 of oogenesis, thus suggesting that Sec61β does not cause a general defect in protein trafficking.

### Sec61β is not required for ER translocation

Data obtained from immunofluorescence analysis in oocytes so far suggest that Sec61β was involved in the transport of the Gurken protein to the plasma membrane, however it was not clear which step of the transport process is affected. We wanted to further characterise this defect and to test the actual process of ER translocation using HeLa cells. Previous studies have also utilized the mammalian cell cultures system for investigating trafficking of EGFR ligands. Gurken and other EGF ligands such as Spitz and Keren can be expressed and secreted in mammalian cells. The process requires Star for ER exit and Rho for proteolytic processing, thus in part recapitulating the trafficking of EGFR ligands as it occurs in the fly system [17,18]. We used this system to examine if changes in the amount of Sec61β could affect the translocation of Gurken into the ER.

**Figure 5:**
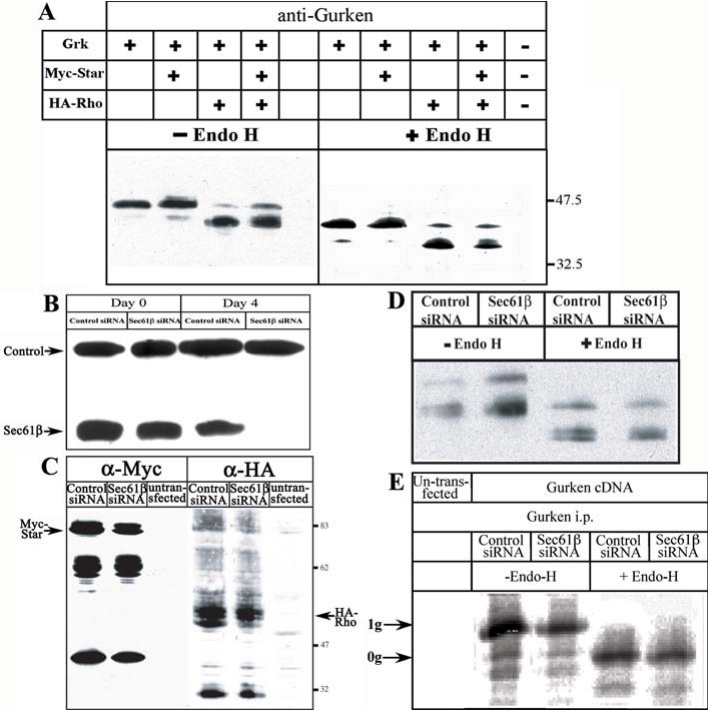
**Expression of Gurken in HeLa cells**. A) Plasmids encoding Gurken protein alone or in combination with Myc tagged Star and/or HA tagged Rho were transfected into HeLa cells as indicated on top of the figure or left un-transfected. Aliquots of the cell lysates were applied to SDS-PAGE either directly or after being treated with EndoH. Western bloting was done using an antibody against Gurken. The numbers on the right indicate the molecular mass in kilo Daltons. B-E) HeLa cells were transfected with plasmid generating a double stranded RNA against Sec61β or a control plasmid together with Gurken, Myc-Star and HA-Rho encoding plasmids as indicated. Cells lysates were applied to SDS-PAGE and western blot and probed with an antibody against Sec61β(B). The lysates were also probed with antibodies against Myc and HA to detect expression of Star and Rho respectively (C). The same sets of cells growing on plates were used for a 15 min pulse with ^35^S methionine and cell lysates were prepared. These lysates were used for western blots with or without EndoH treatment and probed with the Gurken antibody (D). The lysates after the pulse analysis were subjected to immuno-precipitation using anti-Gurken antibody and immuno-precipitated samples were applied to SDS-PAGE either directly or after being treated with EndoH (E). Single glycosylated (0 g) and nonglycosylated (1 g) forms of Gurken are indicated.

Gurken was transfected into HeLa cells, either alone or co-transfected with the regulatory proteins, Star and Rho. On western blot analysis of the whole cell lysate, Gurken is seen to migrate as a 45–47 KDa protein (Fig. 5A). Upon co-transfection with Myc-tagged Star the position of the band is unchanged. When HA-tagged Rho is transfected, Gurken shows faster migration, which most likely represents the intra-membrane cleaved form of Gurken. Gurken is glycosylated as inferred from its sensitivity to Endo-glycosidase H, EndoH (Fig. 5A, right panel). Sensitivity to EndoH is retained even when Star and Rho are transfected along with Gurken indicating retention in the ER. Based on this data we think in this cell type Star does not cause an extensive Gurken export from the ER and Rho is able to process the Gurken within the ER. Expression of Star and Rhomboid was confirmed by western blot analysis using the anti-Myc and anti-HA antibody respectively (data not shown).

In order to investigate Gurken translocation into the ER in the absence of Sec61β, a plasmid was transfected into HeLa cells that was designed to generate double stranded RNA oligo against Sec61β mRNA. As a control, a plasmid designed to generate a scrambled double stranded RNA oligo was also transfected. Three days after the initial transfection the cells were re-transfected with the Sec61β siRNA plasmid or the control siRNA plasmid, together with the expression plasmids for Gurken, Star and Rho. One day after the second round of transfections (and four days after the initial round of transfection) Sec61β protein is no longer detected by western blotting in cells transfected with the Sec61β siRNA plasmid, whereas cells transfected with the control plasmid retain normal level of Sec61β protein (Fig. 5B). Expression of Star and Rho is confirmed by western blot analysis using the anti-Myc and anti-HA antibodies respectively (Fig 5C).

ER translocation of Gurken was investigated as follows: a pulse analysis using ^35^S-methionine was performed and the cells were harvested for immuno-precipitation and western blotting. The actual process of translocation and availability of Gurken for post-translocational processing was determined by the extent of glycosylated protein. Correctly translocated Gurken would be glycosylated in the ER, on the other hand, conditions affecting translocation or downstream events would result in a species of Gurken that is unavailable for glycosylation.

The cell lysates after ^35^S-methionine pulse were probed with antibody against the Gurken protein (Fig 5D). Gurken is visible in the processed, faster migrating form in control cell lysates and lysates with depeleted Sec61β protein. Gurken is EndoH sensitive in cells with normal and reduced Sec61β protein, hence glycosylated and present in the ER (Fig 5D). Guken protein was immuno-precipitated from control and Sec61β depleted cells; half of the immuno-precipitated sample was treated with EndoH and the other half was left untreated, both the samples were applied to SDS-PAGE. We observe that in lanes representing control and Sec61β depleted cells without EndoH treatment, the majority of Gurken protein runs as a single band along with minor lower molecular weight bands. (Fig. 5E, left half). Majority of the Gurken protein is also glycosylated since after EndoH treatment it runs at lower molecular weight (Fig. 5E, right half). It is important to note that the relative ratios of the glycosylated and unglycosylated forms of Gurken protein (1 g and 0 g respectively) does not change in the absence of Sec61β. Thus, the translocation of Gurken into the ER and post-translocational processes such as glycoslation are not affected by the reduced level of Sec61β. These experiments also show that cleavage of Gurken is unaffected by reduced amounts of Sec61β, suggesting that Rho is translocated and functional (Fig 5D).

Taking together the data from co-staining in the oocytes and experiments in HeLa cells it seems that Gurken translocation into the ER and its post-translational modifications remain unaffected by lack of Sec61β. Sec61β seems to affect a step beyond the process of ER translocation.

### Gurken is transported till the Golgi complex in absence of Sec61β

**Figure 6:**
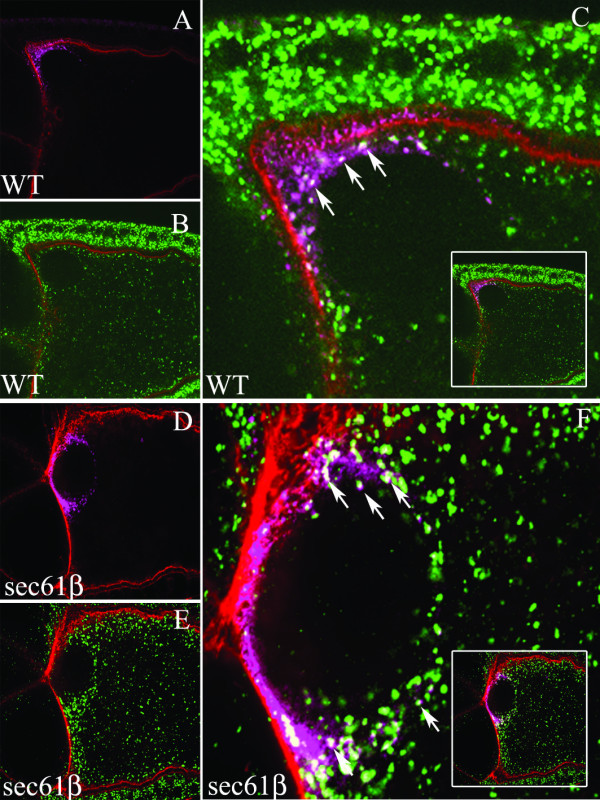
**Co-localization of Gurken with Lva**. Wild type egg chambers and egg chambers derived from germline clones of *sec61β*^*P1 *^were stained with Gurken (Magenta), Lva (green) and Phalloindin (red). A-C) Wild type egg chambers with Gurken localized to the anterior-dorsal region of the ooctyte and also along the plasma membrane (A) and Lva localized in the cytoplasm of the oocyte (B). Co-staining of Gurken shows frequent overlap of the signal for Gurken and Lva, as indicated by the arrows (C). D-F) Egg chambers derived from the germline clones of *sec61β*^*P1 *^with Gurken localized to the anterior-dorsal end of the oocyte (D) and Lva localized in the cytoplasm of the oocyte (E). Co-staining of Gurken shows frequent overlap of the signal for Gurken and Lva, as indicated by the arrows (F). Insets show whole of the co-stained oocyte.

Data obtained from immunofluorescence analysis in oocytes and in HeLa cells suggested that Sec61β was not involved in the transport of the Gurken protein into the ER, on the contrary it appeared that it is required at a later, post-ER step of secretion. The finding that lack of a component of the translocation channel could affect a step beyond the ER translocation was indeed surprising. In order to investigate this further and we wanted to analyse the sub-cellular localization of Gurken in greater detail. In both wild type and mutant oocytes the cytoplasmic pool of Gurken protein is present in the peri-nuclear region and is organised into punctate structures. These Gurken containing punctate structures are interspaced within the dispersed ER in both wild type and sec61β mutant oocytes. In order to establish the identity of these punctuate structures we used antibody against the Lava Lamp (Lva) protein, a cis-Golgi marker [19] together with the Gurken antibody for immunofluorescence analysis. We observed that the punctate structures containing the Gurken protein frequently also contained the Lva protein and this was visible in either the wild type (Fig. 6A–C) or the *sec61β*^*P1 *^oocytes (Fig. 6D–F).

**Figure 7:**
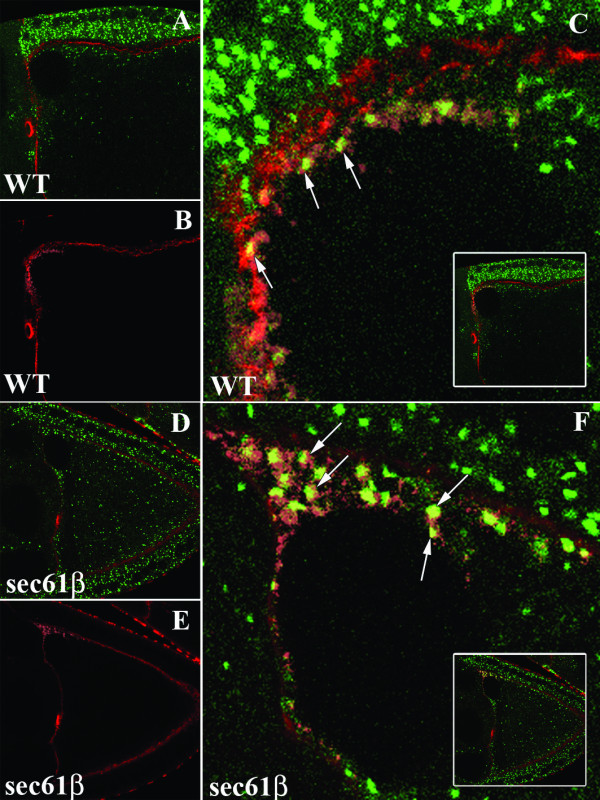
**Co-localization of Gurken with a Golgi Marker**. Wild type egg chambers and egg chambers derived from germline clones of *sec61β*^*P1 *^were stained with Gurken (Magenta) and Phalloidin (red). The ubiquitously expressed Golgi complex marker is in green. A-C) Wild type egg chambers expressing the Golgi marker (A) and stained with Gurken, which is localized to the anterior-dorsal region of the oocyte (B). A magnification of the anterior-dorsal region of the oocyte showing frequent co-localization of punctuate Gurken containing structures with the Golgi marker (C). The inset is same as C but shows the whole egg chamber. D-F) Egg chambers mutant for sec61β expressing the Golgi marker (D) and stained with Gurken, which is localized to the anterior-dorsal region of the oocyte (E). A magnification of the anterior-dorsal region of the oocyte showing frequent co-localization of punctuate Gurken containing structures with the Golgi marker (F). The inset is same as F but shows the whole egg chamber. Co-localization is indicated by white arrows.

We also analysed the sub-cellular localization of the Gurken protein using a ubiquitously expressing an EYFP tagged Golgi marker [20]. In both wild type (Fig 7A–C) and sec61β mutant oocytes (Fig 7D–F) we observed that a significant number of the punctate Gurken structures in the cytoplasm co-localized with the Golgi marker.

This suggests that in the sec61β mutants Gurken protein is able to enter the secretory pathway and traffic till cis-Golgi, further transport to the plasma membrane however is impeded.

### Sec5 retains plasma membrane localization in *sec61β*^*P1 *^germline clones

Based on the data obtained so far it seemed that Sec61β is involved in a post-translocation step during trafficking of a subset of proteins to the plasma membrane. We therefore wanted to analyse the possible post-translocational steps in Gurken transport that could be affected by lack of Sec61β. Recent data suggests that Sec61β interacts with the components of the exocyst machinery in *S. cerevisiae *and in mammalian cells [21,22]. The exocyst complex is a multi-protein vesicle-tethering complex localized at the plasma membrane that plays a role in polarised protein transport [23]. Directional transport of Gurken to the plasma membrane requires the exocyst complex. Germline clones of a hypomorphic allele of a subunit of the exocyst complex, sec5^E13^, shows lack of Gurken at the plasma membrane and cytoplasmic accumulation of Gurken [24]. Based on the interaction data and defects in Gurken localization in germline clones of sec5^E13 ^and *sec61β*^*P1*^, we considered the possibility that failure of Gurken transport from the cytoplasmic location to the plasma membrane in Sec61β mutant egg chambers is due to lack of a functional exocyst.

**Figure 8:**
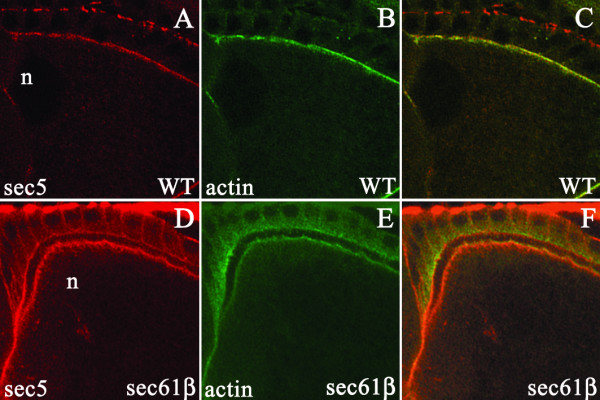
**Localization of Sec5 in stage 10 oocytes**. A-F) Wild type egg chambers or egg chambers from *sec61β*^*P1 *^germline clones were stained with Sec5 (red) and Phalloidin (green). In wild type egg chambers (A) or from *sec61β*^*P1 *^germline clones (D) Sec5 is at a peripheral location in the oocyte. Co-staining with phalloidin (B and E) shows that both in wild type oocytes and oocytes from the germline clones Sec5 co-localizes at the plasma membrane. n indicates the position of the nucleus (C and F).

In order to investigate this possibility we examined the localization of Sec5 protein in germline clones of *sec61β*^*P1*^. We used an antibody against Sec5 to stain the egg chambers during stage 10 of oogenesis. In wild type oocytes Sec5 is localized to the plasma membrane (Fig. 8A–C). In the oocytes from *sec61β*^*P1 *^germline clones (Fig. 8D–E), the localization of Sec5 appears identical to the wild type oocyte. Thus, in absence of Sec61β, Sec5 retains its plasma membrane localization.

## Discussion

### Sec61β 's enigmatic presence in the translocation channel

The mechanism of translocation of proteins across the ER membrane through the Sec61 channel and the function of Sec61β in the translocation process has previously been investigated using reconstituted liposomes, isolated rough microsomes and *S. cerevisiae *system [2,25,26]. These studies suggest that for the mechanistic process of protein translocation Sec61β is dispensable. These data are also consistent with the crystal structure of the Sec61 complex, the α subunit of the complex forms the actual protein conducting channel whereas the β and the γ subunits are associated on the periphery [1]. Hence the question we wanted to address was: if Sec61β is not involved in the process of ER translocation how could we explain its essential role in *Drosophila*.

Major shortcomings in the previous studies investigating the function of Sec61β have been use of limited set of substrates for monitoring protein translocation and use of *in-vitro *models. In the present study we investigated *in-vivo *the molecular function of Sec61β. We report that Sec61β is essential for the plasma membrane transport of the TGFα-like growth factor, Gurken, in *Drosophila *oocytes. Reduced Gurken signalling in these oocytes results in embryonic lethality with severe defects in anterio-posterior axis determination. The surprising observation made in our study was that the defect in Gurken transport to the plasma membrane occurs not during ER translocation but during a later step in the transport process.

### Sec61β affects post-ER transport of Gurken to the plasma membrane

Having observed that the Gurken transport to the plasma membrane is affected in sec61β mutant oocytes we wanted to determine the step in the transport pathway that is affected by the absence of Sec61β. In the first set of experiments we investigated the possible role of Sec61β in ER translocation of Gurken using mammalian cells in culture. The mammalian cell system has been previously characterised to recapitulate the trafficking of EGFR ligands like Gurken and Spitz [17,18]. Gurken is a transmembrane protein with a signal sequence and would require the Sec61 channel for the ER translocation. The ER translocation of Gurken, as determined by comparing the relative amounts of glycosylated and un-glycosylated ^35^S Methionine labelled protein, was unaffected by the absence of Sec61β. We also observed that post-translocational events co-ordinated with translocation, such as glycosylation, remain unperturbed by the absence of Sec61β. Hence we confirm the previous observations that Sec61β indeed does not affect the ER translocation of Gurken. We also observed that the proteolytic processing of Gurken by Rho occurred even in the absence of Sec61β, suggesting an ER translocated and functional Rho.

Immuno-fluorescence data from oocytes revealed the cytosolic fraction of the Gurken protein in both wild type and sec61β mutant oocytes was intermingled with ER, as concluded from the co-staining with Boca, whereas the punctate Gurken structures contain the Golgi marker Lva. Similar results were obtained when Gurken was co-localized with Golgi marker, *p{sqh-EYFP-Golgi}*. Thus in the sec61β mutant oocytes Gurken is able to traffic till the Golgi complex, however further transport to the plasma membrane is impeded. The cytoplasmic punctuate organization of Gurken has been reported previously and shown to also contain dCOG, a component of the complex that tethers ER-derived vesicles to the Golgi membrane [27]. It is important to note that presence of the signal sequence suggests that Gurken most likely is co-translationally translocated into the ER [2]. If a delay in translocation due to lack of Sec61β was to occur this would result in concomitant delay in translation and would result in highly reduced amounts of the Gurken protein in the oocytes. In our studies the amount of protein in the wild type and mutant oocyte does not appear to be drastically different, thus providing additional evidence for normal ER translocation of Gurken in the absence of Sec61β.

Based on our data we think that Gurken transport to the plasma membrane occurs in two distinct steps. The first step is the translation of the localized Gurken mRNA followed by translocation into the ER and transport into the secretory pathway. Gurken is transported along the secretory pathway until it reaches the Golgi complex. This part of the Gurken transport process occurs independent of Sec61β. The second phase in the Gurken secretion is transport of Gurken from Golgi to the plasma membrane, which our results indicate, requires Sec61β.

As mentioned previously, there are two rounds of Gurken signalling during oogenesis. The first round of Gurken signalling occurs during the stage 7 of oogenesis, where Gurken signalling to the posterior follicle cells causes them to take the posterior fate. The follicle cells reciprocate the Gurken signalling from the oocyte by sending an as yet unknown signal back to the oocyte resulting in repolarization of the microtubule skeleton within the oocyte. This event is necessary for the migration of the nucleus to the anterior portion of the oocyte towards the later stages (stage 9) of oogenesis [28]. Although we observed the defect in the Gurken trafficking during the early stages of oogenesis we did not investigate the fate of the posterior follicle cells in the *sec61β*^*P1 *^mutant oocytes. However, the migration of the nucleus to the anterior end of the oocyte was not affected, leading us to conclude that defect in Gurken trafficking at this stage does not result in a significant phenotype.

### Role of Sec61β beyond ER translocation during Gurken Transport

A hint at the post-ER function of Sec61β comes from two independent studies in mammalian cells in culture and in yeast. These studies report that Sec61β could physically and genetically interact with the members of the exocyst complex [21,22]. Exocyst is a complex of eight proteins that associate with the plasma membrane and localise to the regions of membrane addition [23]. In addition it has also been reported that Gurken transport to the plasma membrane requires the exocyst complex [24]. Germline clones of a hypomorphic allele of Sec5 (*sec5*^*E13*^), a subunit of the exocyst complex, results in accumulation of Gurken in the cytoplasm [24]. Thus, it is possible that Gurken transport requires the Sec61β-exocyst interaction and in absence of either of the two components Gurken is no longer transported to the plasma membrane. Various studies have indeed identified components of the exocyst at the ER, the interaction being cell and substrate dependent [21,29]. We did not observe changes in the plasma membrane localization of Sec5 or presence of Sec5 at the ER in sec61β mutant oocytes. We believe that this is presumably due to the transient nature of the interactions and due to the fact that the exocyst contains sub-complexes that actually disassemble from the plasma membrane and Sec5 may not be part of this complex in the oocytes [30].

### Sec61β required for a specific transport step

The critical role of Gurken in development has mandated several regulatory steps in biosynthesis. The highly polarized Gurken transport process begins by localization of the Gurken mRNA at the anterior-dorsal end of the oocyte; this step is necessary but not sufficient to achieve polar transport [28]. Local translation of Gurken at the anterior-dorsal end of the oocyte translocates it into a ER that spans the entire oocyte. Mechanisms exit in the secretory pathway to prevent diffusion of the Gurken protein during its transport from the extended ER to the plasma membrane; these include selection of defined ER exit sites, specific interactions with specific cargo receptors and interactions with the exocyst [24,27,31].

Transport along the secretory pathway involves export of Gurken protein from the ER a process mediated by Cornichon [10]. In our study we observe that even in the absense of Sec61β Gurken could be exported to the Golgi. This indicates that Cornichon is functional in the oocytes. This data also suggests that Star, another ER export factor, is also functional in these ovaries [18,32]. It is important to note this since both these proteins are transmembrane proteins and the fact that they are able to mediate Gurken export out of the ER and hence are functional which indicates that Sec61β does not affect the ER translocation of Star or Cornichon. The next step in the Gurken secretion process is its cleavage by the protease Rho [17,18]. While we could not analyse the cleavage state of Gurken in sec61β mutant ovaries, data from experiments in cultured cells show that even in the absence of Sec61β Rho is indeed able to cleave Gurken. In this system we also observe that the majority of the Gurken protein retains the ER specific glycosylation indicating continued retention in the ER. It is, however, still a substrate for Rho dependent cleavage. Processing of Gurken in the ER has previously been observed [31].

In mediating the traffic of different membrane proteins Sec61β seems to have a specific role in Gurken transport. The specificity of the role is illustrated by the observation that Yolkless does not require Sec61β for plasma membrane transport [24]. The role of Sec61β can be compared to that of Cornichon for Gurken transport and Boca for traffic of the LDL family of proteins. Both these protein like Sec61β are needed for the correct transport of individual proteins and appear to be present at an earlier stage of secretion. Gurken is retained in the oocyte in cornichon mutants although vitellogenesis proceeds normally [10]. Boca on the other hand is required for the trafficking of Yolkless and other LDL receptor family proteins but does not influence Gurken traffic [15]. All these factors are highly specific for certain proteins and do not seem to affect the general transport machinery. Although the role of Sec61β in Gurken transport seem to be indeed specific, we cannot completely rule out the possibility of an indirect effect where Sec61β affects translocation of a factor that is required in Golgi to plasma membrane transport of Gurken.

In development of an organism inter-cellular signalling occurs primarily by the interaction of transmembrane or secreted ligands with transmembrane receptors. Signalling is also initiated when secreted molecules interact with receptors at a considerable distance from the source of the secreted ligand. Therefore precise intra-cellular trafficking of secreted molecules, ligands and receptors is essential for proper signalling and thus development of the tissue. Signalling by the EGFR ligands is an example illustrating regulation during secretion. A detailed study is required to determine the exact role of Sec61β in secretion of signalling molecules, however what seems to be clear is that the function may not be restricted to the ER. In addition, as demonstrated in the present study, Sec61β appears to provide a fine-tuning in trafficking of specific ligands. This study also illustrates that use of a sensitive in-vivo assay like Gurken signalling in addition to in-vitro systems can provide greater insight into the process of protein translocation.

## Conclusion

The Sec61 channel is the only known protein translocation channel in the ER and is conserved in evolution. In this study we have established the role of the β subunit of the channel during plasma membrane traffic of the TGFα-like growth factor, Gurken, during Drosophila oogenesis. Gurken signalling is responsible for early patterning in the embryo, absence of Sec61β and the subsequent defects in Gurken trafficking result in dorsalization of the embryo due to reduced Gurken signalling. We also made the surprising observation that although Sec61β is part of the protein transport channel at the ER it affects the transport of Gurken from the Golgi to the plasma membrane. In addition our study provides an excellent *in-vivo *tool to investigate the process of ER translocation.

## Methods

### Drosophila stocks and phenotypic analysis

The following stocks were used in our experiments:

*y, w hs-FLP; FRT42B ovo*^*D(2R)*^/*CyO*

*y, w;FRT42B sec61β*^*P1*^/*Cyo*

The Golgi Marker *p{sqh-EYFP-Golgi}3 *as described in[20]from the Bloomington Stock Center. To generate germline clones, *y, w;FRT42 sec61β*^*P1*^*/Cyo *females were crossed with *y, w hs-FLP; FRT42B ovo*^*D(2R)*^/*CyO *males. The progeny were heat-shocked at 37°C for 1 hour during the early pupal stage. Ovaries were dissected from females with the genotype *y w hs-FLP; FRT42 sec61β*^*P1*^/*FRT42 ovo*^*D(2R)*^.

### In-situ Hybridization

Ovaries from 1- to 4-day-old females were used for in-situ hybridization with full length, dioxyginin-labeled DNA probe against the Gurken mRNA as described [6].

### Immunocytochemistry and microscopy

Ovaries from 1- to 4-day-old females were dissected in PBS, and kept on ice. Ovaries were fixed in 6:1 Heptane:FIX (Fix: 4% Formaldehyde in PBS) for 15 minutes. All antibody staining was carried out in PBS, containing 0.5% BSA, 0.1% Triton-X-100 and 5% normal goat serum. The following stains and primary antibodies were used: Texas Red-X/FITC phalloidin, mouse anti-Gurken 1D12 (Hybridoma bank), mouse anti-Sec5 22A2 [24], rat anti-Yolkless [16], Anti Boca [15], anti-Lava lamp [19]. Secondary antibodies used were FITC, Cy3 or Cy5 conjugated (Jackson Laboratories). Confocal data were acquired as single images or image stacks of multi-tracked, separate channels with a Zeiss LSM 510 microscope.

### Design of siRNA Constructs in pSupressor

Sec61β siRNAs were designed and cloned into pSupressor as described in the manufacturer's protocols (Imgenex, San Diego).

The Sec61β oligo used was: TGTTCCAGTATTGGTTATGA

A scrambled sequence was used as a control: TTAATTTCTGGGAGCTTATG The sequence is identical to both human Sec61β genes, at the 5' untranslated region. The oligo contained an intervening sequence that forms the stem-loop structure in the RNA and includes a ScaI site for identification of recombinants. The oligo pair was annealed at 20 μM in annealing buffer (100 mM potassium acetate, 30 mM HEPES-KOH (pH 7.4), 2 mM magnesium acetate) at 95°C for 4 min, followed by incubation at 70°C for 10 min and slow cooling to room temperature. Forty picomoles of annealed oligos were phosphorylated by T4 polynucleotide kinase before they were ligated into pSupressor vector digested by SalI and XbaI.

### Transfection and biochemical analysis

HeLa cells were grown in Dulbecco's Modified Eagle's medium (DMEM) supplemented with 10% fetal calf serum and Penicillin and Streptomycin (Invitrogen). Transfections in HeLa cells were done using Lipofectamine-2000 following the manufactures protocol (Invitrogen). 3 μg of each of the plasmid was used for per 10 cm cell culture petriplate containing 70–80% confluent cells. After three days of the first transfection a second transfection was done using the same set of plasmids.

Cells were scrapped off the petriplates and lysis buffer was added [1% Triton, 150 mM NaCl, 5 mM EDTA, supplemented with protease inhibitory cocktail (Roche). SDS-PAGE and western blotting were done following standard protocols. Endoglycosidase H (Endo-H, New England Biolabs) treatment was done as per manufacturer's instructions. Antibodies used for western blotting were rabbit anti-*Drosophila *Sec61β (peptide used corresponds to amino acid 2–10 of the Drosophila sequence, PAPPSSTSV-C), anti-human Sec61β [2], anti-Gurken 1D12 (Hybridoma bank), anti-Myc and anti-HA (Santa Cruz Biotech).

For pulse analysis, cells were depleted of endogenous methionine and cysteine by growing cells in DMEM without methionine and cysteine for two hours before incubation with 100μCi of ^35^S labeled methionine and cysteine per 10 cm petriplate. The cells were pulsed for 10 minutes, washed with PBS, scraped off the plates and lysis buffer was added. Proteins were immuno-precipitated, and applied to SDS-PAGE after treatment with Endo H treatment.

## Authors' contributions

AK conceived the project and did all the experiments. BD supervised the project. All authors read and approved the final manuscript.
